# The epidemic of thyroid cancer in China: Current trends and future prediction

**DOI:** 10.3389/fonc.2022.932729

**Published:** 2022-09-02

**Authors:** Jiayuan Wu, Xiaoyan Zhao, Jianzhong Sun, Chong Cheng, Chunyu Yin, Ruhai Bai

**Affiliations:** ^1^ Clinical Research Service Center, Affiliated Hospital of Guangdong Medical University, Zhanjiang, China; ^2^ Medical Innovation Research Department, People’s Liberation Army (PLA) General Hospital, Beijing, China; ^3^ Health Science Center, Xi’an Jiaotong University, Xi’an, China; ^4^ Hospital of Nanjing University of Science and Technology, Nanjing, China; ^5^ Department of Outpatient, First Medical Center of Chinese People’s Liberation Army (PLA) General Hospital, Beijing, China; ^6^ School of Public Affairs, Nanjing University of Science and Technology, Nanjing, China

**Keywords:** age-period-cohort effect, thyroid cancer, incidence, mortality, projection

## Abstract

**Background:**

Thyroid cancer (TC) is one of the most common cancers in China. The aim of this study was to identify the potential age, period, and cohort effect under the long-term trends in TC incidence and mortality, making projections up to 2030.

**Methods:**

Incidence and mortality data on TC were obtained from the Global Burden of Disease Study 2019. The population predictions were obtained from the United Nations World Population Prospects 2019. An age–period–cohort model was used for the analysis.

**Results:**

From 1990 to 2019, the net drift (the overall annual percentage change of TC over time adjusted for age groups) of the TC incidence was 5.01% (95% confidence interval [CI]: 4.72%, 5.29%) for men and 1.48% (95% CI: 1.14%, 1.82%) for women. The net drift of TC mortality was 1.64% (95% CI: 1.38%, 1.91%) for men and –2.51% (95% CI: –2.77%, –2.26%) for women. Regarding the incidence of TC, both the period and the cohort relative risks (RRs) in men and women showed an overall increasing trend. As to the mortality rate of TC, both the period and cohort RRs in women showed a monotonic declining trend. The period RRs for men decreased after 2015, but the cohort RRs revealed a fluctuating upward pattern. From 2019 to 2030, the TC incidence was projected to rise by 32.4% in men and 13.1% in women, the mortality declining by 13.0% in men and 17.3% in women. The elderly was projected to have an increasing proportion of TC occurrence and deaths.

**Conclusions:**

Over the past 30 years, the incidence rate of TC in China has continually increased, and this trend was projected to continue. Although male mortality has increased in the past, it is expected to decline in the future. The proportion of older people among TC occurrence and death was projected to gradually increase, and the difficulties elderly with TC lrequire more attention.

## Introduction

Thyroid cancer (TC) is the most common endocrine and head-and-neck malignancy, accounting for ∼2.1% of all cancer diagnoses worldwide, and most patients are women, accounting for more than 77.0% of all diagnoses (1). The TC incidence rate has been increasing worldwide since the 1990s, except for Africa where diagnostic technology is limited (2), and the rate is increasing faster than any other malignancy (3). According to Global Cancer Statistics 2018, there were 41,000 new TC deaths worldwide in 2018, accounting for 0.4% of all cancer deaths and ranking sixth in the world for cancer mortality (1). In 2017, the age-standardized incidence rate (ASIR) was 3.2/100,000 globally, and the age-standardized mortality rate (ASMR) was 0.5/100,000 (4).

TC was the cancer with the fourth highest incidence rate in China among its urban population, with annual increases of 14.5% from 2003 to 2007 (5). The TC incidence rate in China was 4.1 per 100,000 people in 2010 (6, 7), which ranks fourth among malignancies in Chinese women after breast, lung, and colorectal cancer (8). In terms of mortality, although the mortality rate of TC has decreased globally in the past 30 years, there was no significant decrease in China (4), and even the mortality rate of TC has increased from 2005 to 2015 (9). As one of the most populous countries in the world, the actual number of TC patients in China could be enormous. Assessing nationwide TC epidemiology is of great significance to evaluate the nationwide TC epidemiology to help promote the formation of relevant policies better (2).

Previous studies have analyzed the long-term incidence and mortality trends of TC in China (9), explored the potential age, period, and cohort effect on TC incidence and mortality trends in several large cities (10), and compared TC incidence trends in China and the United States (11). These studies provided useful information to help understand the TC challenges in China. However, few studies have explored changes in the incidence and mortality rates of TC in different age groups. Furthermore, comprehensive analysis combining past and future trends is still rare. In this study, we used the latest data from the Global Burden of Disease Study (GBD) to analyze the long-term trends of TC incidence and mortality in China. To better understand these trends, we also forecasted the future rate of TC in China. This study is an effective supplement to the existing evidence. The findings from this study may provide important information on policy development and resource allocation to improve the treatment and prevention of TC in China.

## Methods

### Data sources

The data extracted from GBD 2019 were used in this study. GBD 2019 is a large international cooperative project that provides information on more than 300 disease burdens around the world (12). The GBD 2019 data for China can mostly track back to two sources: the China Disease Surveillance Points system and the Vital Statistics System. These two systems are well designed and provide a national representation of disease in China (13, 14). Age-standardized incidence and mortality rates were weighted by the GBD 2019 age-standardized population. In this study, TC was defined according to the International Classification of Diseases (ICD10: C73-C73.9, D09.3, D09.8, D34-D34.9, D44.0; ICD9: 193-193.9, 226-226.9) (12).

The population predictions for China were obtained from the United Nations World Population Prospects 2019 and were used to estimate China’s population after 2019.

### Statistical analyses

An age–period–cohort (APC) framework was used to evaluate the effects of age, period, and cohort on disease-rate outcomes. The equation can be expressed specifically as follows (15):


$$Y=\text{log}\left(M\right)=\mu +\alpha Age_{i}+\beta Period_{j}+\gamma Cohort_{k}+\epsilon$$

where *M* indicates the incidence or mortality rate of the corresponding age group; within the APC model α, *β*, and γ are the coefficients for age, period, and cohort effect, respectively, μ is the intercept, and *ϵ* is the random error of the model.

The following functions were mainly focused in this study: net drift, which indicates the overall annual percentage change over time adjusted for age groups; local drift, which indicates the annual percentage change within each age group; longitudinal age curve, which represents the age effect, indicates the different risks for different age groups; period relative risks (RRs), which represent the period effect, reflect the different disease risks for different time period; and cohort RRs, which represent the cohort effect, reflects different disease risks in different birth cohorts (16).

To conduct the APC frameworks, incidence cases, mortality cases, and population data were arranged into consecutive 5-year periods from 1990–1994 (median 1992) to 2015–2019 (median 2017), successive 5-year age groups from 20–25 years to 90–95 years, and 20 consecutive birth cohorts ranging from 1898–1992 to 1993–1997. The webtool from the United States National Cancer Institute was used to obtain these parameters (17). By default, the reference points were median age group (55–59 years), period (2000–2004), and birth cohort (1943–1947). This study used Wald chi-square tests to calculate the significance of these estimated parameters and functions. All statistical tests were two-sided, and P values lower than 0.05 were considered statistically significant.

The Bayesian APC method was used to forecast the future rates and number of cases of TC from 2020 to 2030, which has a high prediction coverage (18). Age, period, and cohort effects were modeled using a random walk of second order. The Bayesian APC models were developed using the “BAPC” package. BAPC uses integrated nested Laplace approximations for full Bayesian inference, which would help avoid any convergence and mixing issues introduced by Markov chain Monte Carlo sampling techniques traditionally used in the Bayesian approach. R version 4.0.5 was used to perform statistical analyses.

## Results

### Net drift and local drift values for TC incidence and mortality

**Figure 1:**
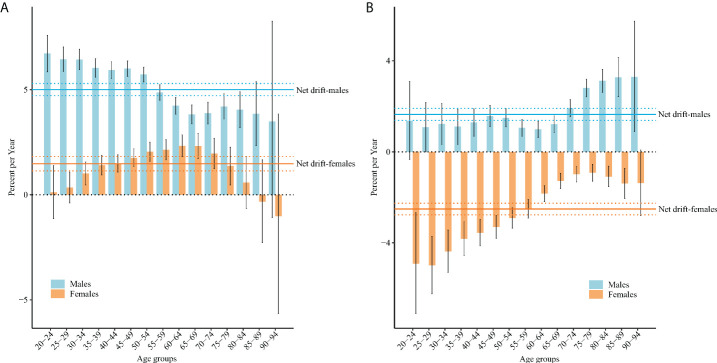
Local drift with net drift values for TC incidence **(A)** and mortality **(B)** in China. Age-group-specific annual percentage change (local drift) and the overall annual percentage change (net drift) of TC incidence and mortality rates, and the corresponding 95% CIs.

**Figure 1** displays net drifts, which represent annual percentage changes overall, and local drifts representing these changes in different age groups. The net drift of the TC incidence was 5.01% (95% CI: 4.72% to 5.29%) per year for men and 1.48% (95% CI: 1.14% to 1.82%) per year for women. The net drift of TC mortality was 1.64% (95% CI: 1.38% to 1.91%) per year for men and –2.51% (95% CI: –2.77% to –2.26%) per year for women.

For incidence, the local drifts for men aged 20–89 years and women aged 30–79 years were both higher than 0 (both P< 0.05). The largest local drift value for men was 6.72% (95% CI = 5.85% to 7.59%) in those aged 20–24 years, and for women it was 2.33% (95% CI = 1.81% to 2.85%) in those aged 60–64 years. For mortality, the local drifts for men aged 30–94 years were higher than 0 (both P< 0.05), and those for women aged 20–89 years were lower than 0 (both P< 0.05). The largest local drift value for men was 3.29% (95% CI = 0.90% to 5.74%) in those aged 90–94 years, and for women it was –4.99% (95% CI = –6.24% to –3.71%) in those aged 25–29 years.

### Longitudinal age curves of TC incidence and mortality by sex

**Figure 2:**
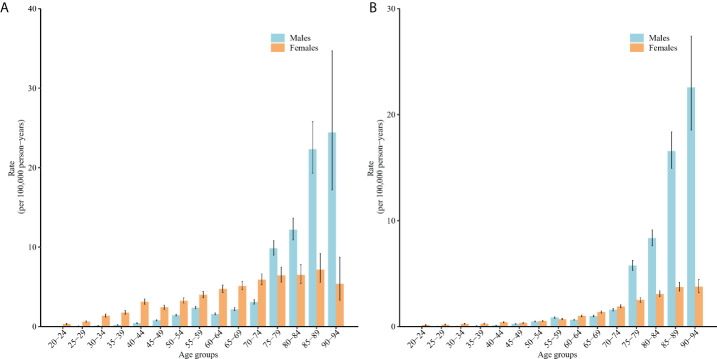
Longitudinal age curves of TC incidence **(A)** and mortality **(B)** in China. Fitted longitudinal age-specific rates of TC incidence and mortality (per 100,000 person-years) and the corresponding 95% CIs (some of these were too narrow to display in the figure).

**Figure 2** displays the longitudinal age curves of incidence and mortality. For men, the risks of TC incidence and mortality exhibited an accelerating growth after 75 years of age. For women, the risks of TC incidence increased slightly and fluctuated and began to decrease slightly after 90 years of age; there was an overall increase in the risks of TC mortality.

### Period and cohort RRs of TC incidence and mortality rates by sex

**Figure 3:**
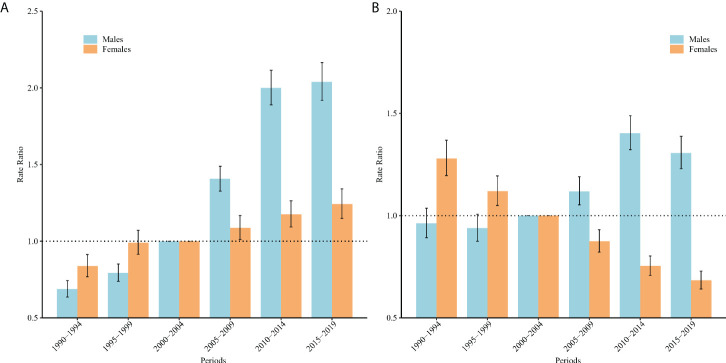
Period relative risks (RRs) of TC incidence **(A)** and mortality **(B)** rates by sex in China. The RRs of each period compared with the reference period (from 2000 to 2004) adjusted for age and non-linear cohort effects, and the corresponding 95% CIs.

**Figure 3** displays the estimated period RRs of incidence and mortality. For men, the period RRs of incidence and mortality increased gradually during the period of 1990–1994 and 2010–2014, but this trend slowed down for incidence and sharply decreased for mortality after 2010–2014 periods. For women, the period RRs for incidence increased slightly and monotonically, but those for mortality decreased sharply.

**Figure 4:**
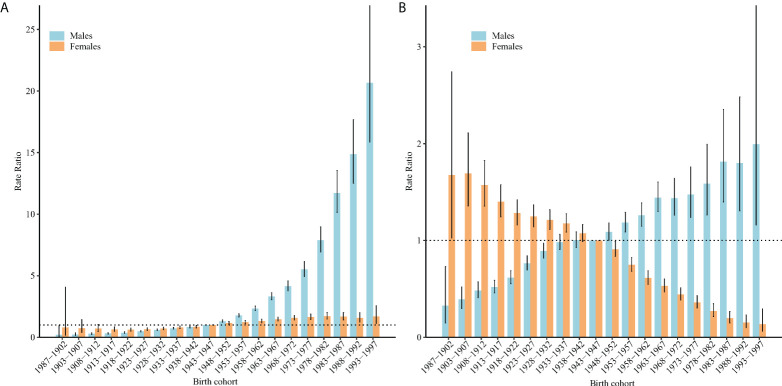
Cohort RRs of TC incidence **(A)** and mortality **(B)** rates by sex in China. The RRs of each cohort compared with the reference cohort (birth cohort 1943–1947) adjusted for age and non-linear period effects, and the corresponding 95% CIs.

**Figure 4** displays the estimated cohort RRs of incidence and mortality. For men, the cohort RRs of incidence increased exponentially, and the cohort RRs of mortality exhibited a stable increase with some small deviations. For women, the cohort RRs of incidence increased with some small deviations, while there was a downward trend for the cohort RRs of mortality. Furthermore, the results of Wald chi-square tests indicated that all these functions (net drifts, local drifts, cohort effects, and period effects) showed a statistically significant on both incidence and mortality (P< 0.05 for all).

### TC incidence and mortality projection

**Figure 5:**
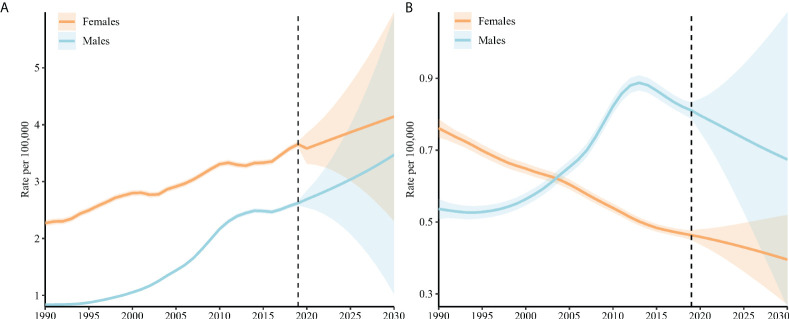
TC incidence **(A)** and mortality **(B)** rates in China (aged 20–94 years). The fan shows the predictive distribution between the 5% and 95% quantiles. The predictive median is shown as solid red line. The vertical dashed line indicates where prediction started. Age-standardized incidence and mortality rates were weighted by the GBD 2019 age-standardized population.

**Figure 6:**
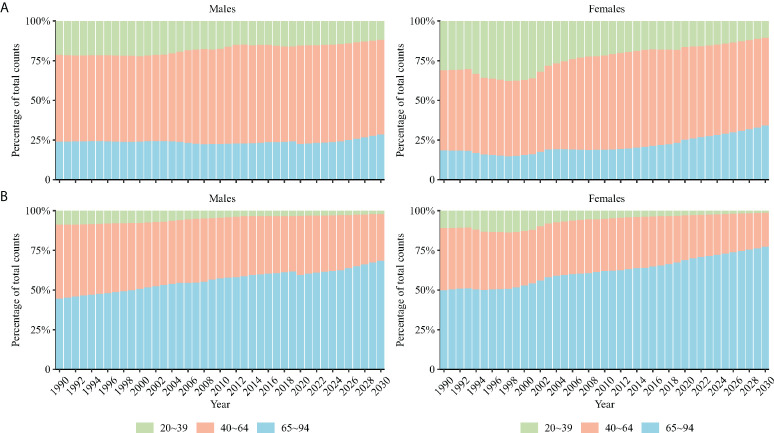
Percentage of modeled and projected counts occurring **(A)** and death **(B)** from TC among people in three age groups (20–39, 40–64, and 65–94).

**Figure 5** displays the projected rates of TC aged 20–94 years. The TC incidence was projected to rise from 2.62 (95% CI = 2.58 to 2.66) per 100,000 in 2019 to 3.47 (95% CI = 1.02 to 5.92) in 2030 in men (increased by 32.4%), and 3.66 (95% CI = 3.61 to 3.71) to 4.14 (95% CI = 2.31 to 5.98) in men (increased by 13.1%). The TC mortality was projected to decrease from 0.46 (95% CI = 0.45 to 0.48) per 100,000 in 2019 to 0.40 (95% CI = 0.27 to 0.52) in 2030 in women (decreased by 13.0%), and 0.81 (95% CI =0.79 to 0.83) to 0.67 (95% CI =0.26 to 1.08) in men (decreased by 17.3%). The elder was projected to have an increasing proportion of TC occurrence and deaths (**Figure 6**).

## Discussion

This study explored the long-term trends in TC incidence and mortality in China and used the APC framework to examine potential age-, period-, and cohort-specific effects on these trends, predicting the TC rate from 2020 to 2030. For incidence, this study found that the net drift increased for both men and women in China over the past three decades. The period RRs and cohort RRs of TC incidence show an overall increasing trend in both men and women. For mortality, although the net drift indicated overall decreasing trends in women, the net drift in men increased in China over the past three decades. In contrast to the continuous decrease in the period RRs and cohort RRs of mortality in women, the period RRs and cohort RRs of TC mortality continually increased in men. We estimated that by 2030, TC incidence would increase in both men and women from 2019, but the mortality would decrease. The elder were projected to have an increasing proportion of TC occurrence and deaths.

In our study, the incidence rate of TC has increased in China over the last 30 years and was projected to increase in the future. In the past few decades, many countries have experienced an increase in TC burden (4, 19). This increased incidence of TC may be related to the improvement of diagnostic technology (19, 20). With the introduction and widespread use of ultrasound and fine-needle aspiration biopsy in the 1980s, a large number of small papilliform thyroid tumors could be found in patients, which previous technology was unable to detect (19). Increase in medical exposure (e.g., computed tomography) to ionizing radiation from diagnostic imaging may contribute to the increase in TC incidence (21). Previous evidence has proved that computed tomography utilization increased from 9.8% in 2005 to 13.9% in 2008. In Shanghai, In the 12 years before 2008, the annual per capita doses of diagnostic and therapeutic procedures in nuclear medicine doubled (22). The prevalence of obesity in China may also contribute to the increase in TC incidence. Obesity is an important risk factor for TC (19, 23). In the past 40 years, the prevalence of overweight and obese individuals increased rapidly in China (24). In 2014, China’s obese population exceeded that of the United States, and it became the country with the highest prevalence of obesity in the world (25). It is expected that the prevalence of obesity will continue to increase in the future in China (26), a trend that also suggests an increased in the incidence of TC to some extent.

In this study, we found that, unlike for men, the net drift for women’s mortality decreased over the past three decades. This may be related to the fact that women may detect TC earlier due to obstetrics and gynecology examinations during reproductive age (27), which may contribute to decreasing TC mortality to some extent. Although the mortality of TC for men showed an overall increased tendency over the past three decades, this trend was projected not to continue in the future. This reversal trend may reveal potential favorable period or cohort effects.

Previous studies have indicated that age is one of the important diagnostic indicators for predicting benign and malignant thyroid nodules (28). In our study, elderly people had higher TC risks of incidence and mortality, which may be related to the following reasons. First, with progress in treatments, cancer patients survive significantly longer and more often die during relapse of the original tumor or from a new cancer. Second, an increasing number of the elderly go to hospital or die in hospital; causes of death of elderly people, especially from cancer, are therefore now clearer (29). Thirdly, the organs of the elderly deteriorate with age, and their metabolism and tolerance to treatment decrease (30). It was particularly interesting that the present study found that although the risk of TC occurrence and death generally increases with age, this increase is not linear: the risks of TC occurrence and death increase rapidly in men after they reach 70 years of age, which provides a reference for more effective future public health interventions.

The period effect partly demonstrated the direct influence of social factors on disease. Our study indicated that the period effect of TC mortality in men in China continually increased before 2014; this was followed by a significant decline, which showed a positive signal for the burden of TC in men. The decline in mortality risks may be related to the improvement of a series of TC-related medical resource allocation systems. Since 2010, some provinces in China have included cervical ultrasound into regular inspection items for urban workers (2). Early diagnosis and appropriate treatment of TC can improve prognosis and reduce mortality (31). Moreover, as a reaction to past experience, China has released a series of TC-related diagnosis and treatment guides after 2010, including the Guidelines for Diagnosis and Treatment of Thyroid Nodules and Differentiated Thyroid Cancer in 2012 and Expert Consensus on the Diagnosis and Treatment of Small papillary Thyroid Cancer in 2016 (32); within current guidelines for TC, recommendations for initial management generally comprise a combination of surgical treatment, radioactive 131iodine (RAI) therapy for most patients, and thyroid-stimulating hormone (TSH) suppression therapy (33), and the information on TC was reported to the National Central Cancer Registry systems, all of which contribute to reducing the risk of TC mortality.

The cohort effect reflects different disease risks in various birth cohorts (34). This study showed that the risks of TC incidence rate in men and women continually increased as the birth cohort moved forward. As stated above, the improvement of diagnostic technology and the increasing prevalence of TC risk factors (e.g., obesity) all played an important role in increasing the risk of TC incidence. We also found that the cohort effect of TC mortality decreased in women and increased in men as the birth cohort moved forward. Obstetric examinations during a woman’s early reproductive age and continually improving TC examination techniques have reduced the risks of TC mortality in newly born women (27). The increasing risk of TC death in men may be attributed to the rapid growth in TC risk factors (e.g., obesity). Compared to women, men have a greater increase in the amplitude of obesity burden (26). The increasing cohort risks of TC mortality in men provide a useful clue into TC prevention and control in China and remind us that more attention needs to be paid to the burden of TC in men. Interestingly, the increased cohort effect on TC death in men did not affect male mortality in the future; this may be related to the favorable effects (period effect) affecting larger strata of TC mortality than the negative ones (cohort effect).

In this study, the elder was projected to have an increasing proportion of TC occurrence and deaths. China is rapidly transforming into an aging nation due to a decline in fertility rate and increase in life expectancy (35). According to the National Bureau of Statistics of China 2020, the number of people aged 60 years and older reached 253.88 million in 2019, accounting for 18.1% of the total Chinese mainland population. As shown in our study, the elderly have a higher risk of TC occurrence and death; this may somewhat explain the increasing proposition of TC occurrence and death among the elderly.

### Limitations

There were some limitations to the present study. First, the data of TC in this study were estimates at the national macro level; there was an inevitability of ecological fallacy because interpreted population results are not necessarily valid for individuals. Therefore, related hypotheses from this study still need further confirmation in larger-scale, individual-based studies. Second, also GBD has used many steps to enhance the data quality and comparability, the completeness and accuracy of primary data may somehow lead to bias (12). Third, similar to other studies in forecasting, unexpected events in health system or social events may change the trends fundamentally. Fourth, due to data limitations, this study did not analyze the subtypes of TC, which have different biological behaviors with profoundly different mortalities (36). Fifth, this study did not analyze the trends in TC incidence and mortality between China’s urban and rural areas. Considering the rapid increase in burden of TC risk factors in China’s rural population (e.g., obesity) (26), as well as changes in the demographic structure of the rural population (37), analyses of the incidence and mortality trends of TC in China’s urban and rural areas must be conducted independently.

## Conclusions

In summary, the incidence of TC in China has generally increased among both men and women over the last three decades and will continue to increase in the future. Although mortality has increased over the past 30 years in men, this increasing trend was projected not to continue in the future. The proportion of older people among TC occurrence and death was projected to gradually increase. Considering the aging process in China, TC may have a high impact on the health of the Chinese elderly. More effective efforts are needed.

## Data availability statement

Publicly available datasets were analyzed in this study. This data can be found here: http://ghdx.healthdata.org/gbd-2019.

## Ethics statement

The GBD study uses deidentified, aggregated data. Therefore, a waiver of informed consent was reviewed and approved by the University of Washington Institutional Review Board.

## Author contributions

The authors’ responsibilities were as follows: RB and XZ designed the research. RB and JW performed the data-analysis; JW, RB, XZ, and CY drafted the original manuscript; JW, JS, and CC critically revised the manuscript; CY and JW provided administrative support for the project and had primary responsibility for the final manuscript; all authors read and approved the final manuscript.

## Acknowledgments

We are grateful to the individuals who participated in the study.

## Conflict of interest

The authors declare that the research was conducted in the absence of any commercial or financial relationships that could be construed as a potential conflict of interest.

## Publisher’s note

All claims expressed in this article are solely those of the authors and do not necessarily represent those of their affiliated organizations, or those of the publisher, the editors and the reviewers. Any product that may be evaluated in this article, or claim that may be made by its manufacturer, is not guaranteed or endorsed by the publisher.
